# Antimicrobial and Antibiofilm Activities of Essential Oils against *Escherichia coli* O157:H7 and Methicillin-Resistant *Staphylococcus aureus* (MRSA)

**DOI:** 10.3390/antibiotics9110730

**Published:** 2020-10-24

**Authors:** Nicolás Gómez-Sequeda, Marlon Cáceres, Elena E. Stashenko, William Hidalgo, Claudia Ortiz

**Affiliations:** 1 Grupo de Investigación en Bioquímica y Microbiología (GIBIM), Universidad Industrial de Santander, Bucaramanga 680002, Colombia; nigomez33@uan.edu.co (N.G.-S.); marlon2127899@correo.uis.edu.co (M.C.); ortizc@uis.edu.co (C.O.); 2 Escuela de Microbiología, Facultad de Salud, Universidad Industrial de Santander, Bucaramanga 680002, Colombia; elena@tucan.uis.edu.co; 3 Escuela de Química, Facultad de Ciencias, Universidad Industrial de Santander, Bucaramanga 680002, Colombia; 4 Centro de Cromatografía y Espectrometría de Masas, CROM-MASS-CENIVAM, Facultad de Ciencias, Universidad Industrial de Santander, Bucaramanga 680002, Colombia

**Keywords:** essential oil, MRSA, *E. coli* O157:H7, antibiofilm activity, natural antimicrobial compound, *Lippia origanoides*

## Abstract

The emergence of multidrug resistant microorganisms represents a global challenge due to the lack of new effective antimicrobial agents. In this sense, essential oils (EOs) are an alternative to be considered because of their anti-inflammatory, antiviral, antibacterial, and antibiofilm biological activities. Therefore, multiple efforts have been made to consider the potential use of EOs in the treatment of infections which are caused by resistant microorganisms. In this study, 15 EOs of both Colombian and introduced aromatic plants were evaluated against pathogenic strains of *E. coli* O157:H7 and methicillin resistant *Staphylococcus aureus* (MRSA) in planktonic and sessile states in order to identify relevant and promising alternatives for the treatment of microbial infections. Forty different compounds were identified in the 15 EO with nine of them constituted mainly by oxygenated monoterpenes (OM). EOs from *Lippia origanoides*, chemotypes thymol, and carvacrol, displayed the highest antibacterial activity against *E. coli* O157:H7 (MIC_50_ = 0.9 and 0.3 mg/mL, respectively) and MRSA (MIC_50_ = 1.2 and 0.6 mg/mL, respectively). These compounds from EOs had also the highest antibiofilm activity (inhibition percentage > 70.3%). Using scanning electron microscopy (SEM), changes in the size and morphology of both bacteria were observed when they were exposed to sub-inhibitory concentrations of *L. origanoides* EO carvacrol chemotype. EOs from *L. origanoides*, thymol, and carvacrol chemotypes represented a viable alternative for the treatment of microbial infections; however, the Selectivity Index (SI ≤ 3) indicated that it was necessary to study alternatives to reduce its in vitro cytotoxicity.

## 1. Introduction

Antibiotics are one of the main defense weapons in our fight against pathogenic microorganisms; however, the abuse of these drugs in both medical and industrial fields [1,2] have led to the emergence of multidrug resistant pathogenic bacteria which is one of the most serious public health problems of this century [3,4]. Among the bacteria that pose the greatest threat to world public health are both methicillin resistant *Staphylococcus aureus* (MRSA) and Shiga toxin-producing *Escherichia coli* (STEC), with serotype O157:H7, being the most prevalent with 1.2 annual cases per 100,000 inhabitants since 2010 [5,6].

Not only *E. coli* O157:H7 but MRSA are also responsible of the majority of nosocomial and community-associated infections [7,8], occupying the first and second place among the most common pathogens with 15 and 12% of total cases, respectively [9]. They are also commonly associated with outbreaks of foodborne diseases, which can cause a wide range of illnesses, such as endocarditis, osteomyelitis, pneumonia, septicemia, skin infections, food poisoning, and hemolytic uremic syndrome, among others [10,11,12]. 

*E. coli* O157:H7 and MRSA have also ability to adhere on living or inert surfaces [13,14,15], forming amorphous mono or poly microbial structures called biofilm, which is mostly composed of an extracellular polymeric substance of proteins, polysaccharides, nucleic acids, and water [13]. Biofilm constitutes a resistance strategy that prevents the permeability of the drug and increases drug tolerance hundreds of times [16,17,18]. Recent investigations point out that at least 65% of all bacterial infectious diseases and 70% of chronic infections in humans could involve biofilms [19,20].

For this reason, it is necessary to join efforts in the search and development of new antimicrobial agents in order to inhibit or eradicate the formation of bacterial biofilm. In this aspect, the essential oils (EOs) from aromatic plants constitute a viable alternative due to their different modes of action that affect several bacterial cell targets at the same time, decreasing the possibility of the emergence of resistant microorganisms [21]. In particular, EOs are composed primarily of aldehydes, phenols and terpene alcohols that are associated with high antimicrobial activity [22], therefore, they constitute a source of molecules with bactericidal, fungicidal, antiparasitic, and insecticidal activity [23,24]. Food and Drug Administration (FDA) generally considers that many individual components of the EO are safe, which has allowed its use in multiple applications in medical, pharmaceutical, food, cosmetic, and health industry [25,26]. 

Thymol and carvacrol are monoterpenes present mainly in the EO of the *Origanum* genus and some *Lippia origanoides* chemotypes. These metabolites exhibit antibacterial, antifungal, anti-inflammatory, and analgesic activities and therefore, during the last decades, they have been of great interest as candidate molecules for developing pharmaceutical products [27].

Moreover, EOs from plants that are produced in the Chicamocha river canyon in Colombia have shown a significant variation on their main constituents (chemotypes) [28], causing variations in its biological activity and potency [29]. This is because of external factors, such as soil quality and climatic conditions [30]. Due to the vital importance of identifying relevant and promising alternatives for the treatment of microbial infections, in this work, we evaluated 15 EOs corresponding to 12 species of Colombian and foreign aromatic plants against the pathogenic strains MRSA and *E. coli* O157:H7 in both planktonic and sessile states (biofilm).

## 2. Results 

### 2.1. Gas Chromatography-Mass Spectrometry (GC/MS) Analysis

Chemical analysis by GC/MS allowed the identification of 40 different compounds (>0.5%) in the 15 EOs of the plant species under study (Table 1). When examining the relative amount of individual compounds, it was found that oxygenated monoterpenes (OM) were major compounds in nine of the 15 oils, which represented 49% of the total identified compounds, followed by the oxygenated compounds (OC, 20%), Monoterpenic hydrocarbons (MH, 15%), sesquiterpene hydrocarbons (SH, 13%), and oxygenated sesquiterpenes (OS, 3%).

Among OM, geranial and geraniol were the most common compounds in the species of the genus *Cymbopogon*; being geranial (33%) predominant in *C.* (CF) and geraniol (38.7% and 17.8%) in *C. martini* (CM) and *C. nardus* (CN), respectively. These results are comparable to those obtained by other authors, which highlight the remarkable interspecific differences in the main constituents of *Cymbopogon* EOs that are consistent with their morphological variations [31,32]. OM were also the major components in *Swinglea glutinosa* (SG), *Salvia officinalis* (SO), *Tagetes lucida* (TL), and *Rosmarinus officinalis* (RO), among which 1.8-cineole was the most abundant in RO and SG (17.5 % and 36.6%, respectively), while *trans*-tujone (20.4%) and estragole (79.9) were the most representative compound in OS and TL, respectively.

In comparison with the *Cymbopogon* spp, in *Lippia* spp were observed notorious differences in the major components of the species and chemotypes studied. In *Lippia alba*, carvone chemotype (LAC) and *L. alba* citral, chemotype (LAC), predominant OMs were carvone (31.3%) and geranial (27%), respectively. On the other hand, *L. origanoides*, thymol chemotype (LOT) and *L. origanoides*, carvacrol chemotype (LOC) EOs were mostly constituted by OC, being thymol (32.7% and 22.1%) the most abundant, followed by carvacrol (18.8% and 10.7%), respectively. In *L. origanoides* phellandrene chemotype (LOP), MH represented 34% of the identified compounds. These results are comparable to those obtained by Stashenko et al. [27,33]. Thymol (23%), benzyl benzoate (20.8%), and 1-isopropenyl-4-methyl-1-cyclohexane (24.4%) were identified as major compounds in *Thymus vulgaris* (TV), *Cananga odorata* (CO) and *Satureja viminea* (SV), respectively.

Estragole was the most abundant component in the TL oil (79.9%). This molecule was only found in this oil. On the contrary, thymol was the most abundant compound in LOC, LOT, and TV with 32.7%, 22.1%, and 23%, respectively. *trans*-β-Caryophyllene (6.4–11.8%) was the most common substance present in seven of the 15 oils. They were followed in frequency of appearance by *p*-cymene (1.1–20%), 1,8-cineol (5.3–36.6%), and geraniol (6.1–38.7%).

### 2.2. Determination of the Minimum Inhibitory Concentration (MIC) and Minimum Bactericidal Concentration (MBC) of EOs

Antimicrobial activity of EOs determined in vitro against *E. coli* O157:H7 and MRSA, was carried out following the broth microdilution protocol [34,35]. Values of MIC_50_ and MBC of the EOs for both bacteria are presented in Table 2. Four of the 15 oils (CM, LOT, LOC, and TV) had activity against *E. coli* (Gram-negative) and five (CF, LOT, LOC, LOF, and RO) against MRSA (Gram-positive). Among the oils that showed activity against *E. coli* O157:H7 the EO with highest activity was LOC (0.4 mg/mL) and the lowest one was CM oil, which had an activity up to three times lower compared to LOC (1.4 mg/mL). Of the four EOs only three had a MBC < 3 (LOT, LOC, TV), while CM oil had a MBC that of higher than the concentrations tested during the study (>3 mg/mL). Both CM and TV oils did not show any activity against MRSA. On the other hand, a third of the EOs tested in the study presented some activity against MRSA, being LOC again the EO with the highest antimicrobial activity (0.6 mg/mL) and the highest MBC (1.5 mg/mL), which was comparable to that obtained against *E. coli* O157:H7. The other EOs such as CF, LOT, LOF, and RO had a lower antibacterial activity compared to LOC, of which two oils (CF and LOT) had an MBC ≤ 3. It should be noted that only LOC and LOT oils had antibacterial activity against both *E. coli* O157:H7 and MRSA.

### 2.3. Inhibition of Biofilm Formation by EOs

Inhibition of biofilm formation was performed for the 15 oils regardless of the results obtained in the MIC_50_. Biofilm formation was quantified by crystal violet method described. The results showed different effects on the growth of the biofilm, these results are presented in Table 3. Only five of 15 EOs inhibited biofilm formation of *E. coli* O157:H7 and MRSA. EOs from LOT and LOC were the most potent, and inhibited biofilm formation above 70% in both bacteria, with MIBC_50_ values of 0.45 and 0.19 mg/mL in *E. coli*, and 1.6 and 0.7 mg/mL in MRSA. Other EOs such as those from TV (MIBC_50_ = 0.81 mg/mL, 70% inhibition) and CM (MIBC_50_ = 1.12, 55% inhibition) had only an effect on *E. coli*, whereas CF (MIBC_50_ = 1.83 mg/mL, 78.1% inhibition) and CO (MIBC_50_ = 0.57 mg/mL, 83% inhibition) only had an effect on MRSA.

### 2.4. EOs Cytotoxicity Assay in the Vero Cell Line

Based on previous results of MIC_50_, MBC and MIBC_50_ in *E. coli* and MRSA, the best EOs were selected and were there cytotoxicities on the Vero cell line (African green monkey kidney, ATCC No. CCL-81). Therefore, EOs obtained from LOT, LOC, TV, and CM were selected. EOs isolated from LOC and LOT demonstrated higher antibacterial activity in both bacteria, whereas that obtained from CF only showed activity in MRSA and TV in *E. coli*.

**Figure 1:**
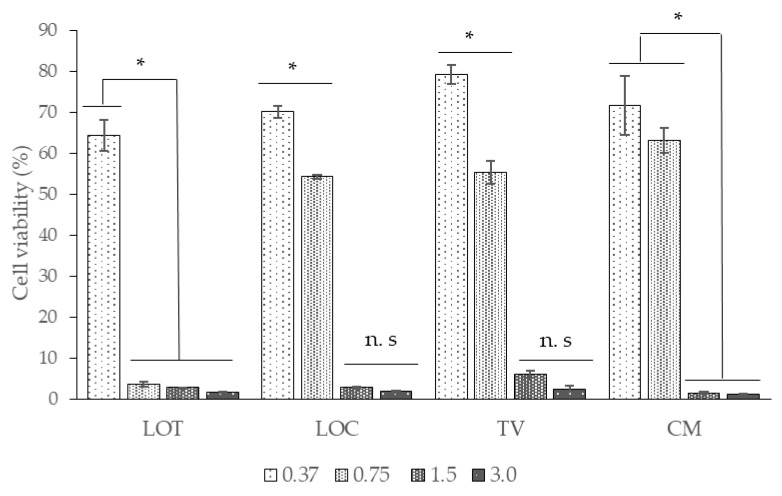
Cell viability assay of Vero cells treated with the EOs obtained from LOT, LOC, TV, and CM. Data are present as the means ± standard deviation. Asterisks indicate significant differences among concentrations of each EO assessed (* *p* < 0.05; n. s, no significant; multiple range test).

These results showed a high cytotoxicity of all EOs at high concentrations (1.5–3 mg/mL) where viability of Vero cells did not exceed 6% (Figure 1). On the other hand, Vero cell viability increased significantly (50–80%) at lower concentrations (0.37–0.75 mg/mL) in most of the EOs (LOC, TV, and CM) except for EO isolated from LOT, where only a good cell viability was obtained (greater than 60%) at the lowest EO concentration (0.37 mg/mL).

To verify if the analyzed EOs have higher antibacterial activity than their cytotoxicities, as expected from a good candidate for antibacterial drug, the Selectivity Index (SI) was evaluated (see Table 4). Compounds with SI ≥ 3 were considered selective, while compounds with SI < 3.0 were considered non-selective [36]. None of the EOs showed SI ≥ 3, and higher SI was observed for EO obtained from LOC against *E coli* O157:H7 (SI = 2.05). However, it should be noted that SI value is commonly used to evaluate pure compounds or drugs and the EOs are complex mixtures of compounds.

### 2.5. Visualization of the Morphological Alterations by Scanning Electron Microscopy (SEM)

**Figure 2:**
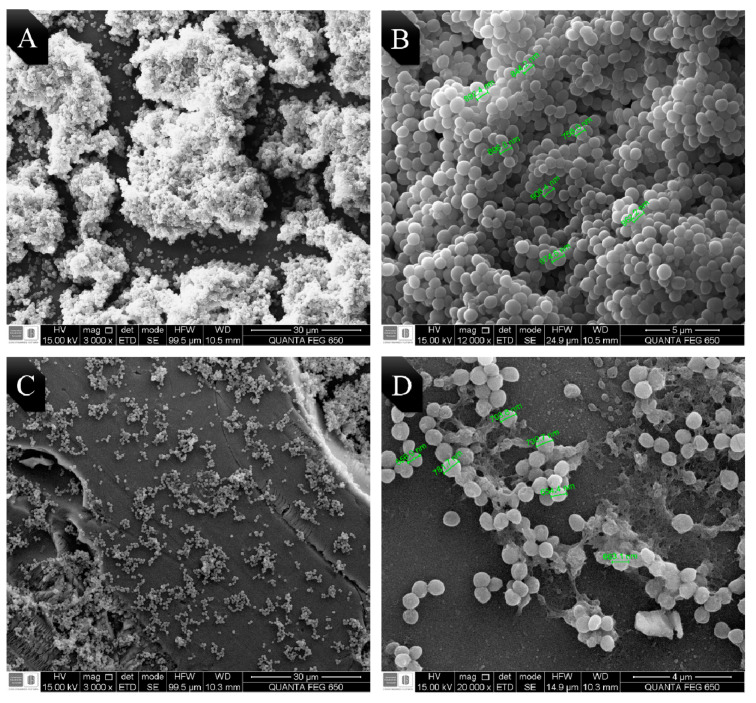
Micrographs of MRSA on frosted glass coupons obtained by SEM. (**A**) Biofilm control of MRSA at 3000×. (**B**) Magnification of the biofilm control at 12,000×. (**C**) MRSA biofilm treated with LOC (0.5 MIC50) at 3000×. (**D**) Magnification of the MRSA biofilm treated with LOC (0.5 MIC50) at 20,000×.

**Figure 3:**
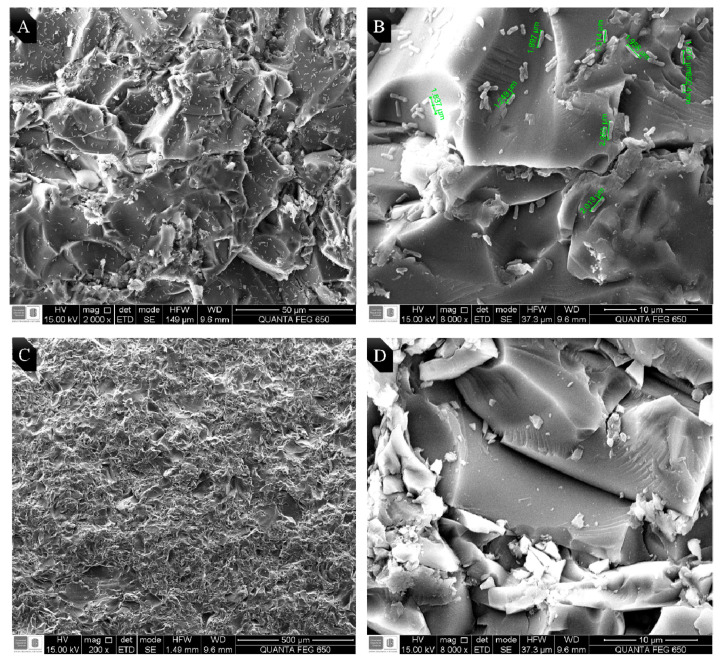
Micrographs of *E. coli* O157:H7 on frosted glass coupons obtained by SEM. (**A**) Control biofilm (2000×). (**B**) Magnification of control biofilm (8000×). (**C**) Biofilm of *E. coli* O157:H7 treated with LOC (0.5 MIC50) (200×). (**D**) Magnification for biofilm treated with EO LOC (0.5 MIC50) (8000×).

EO from LOC was chosen due to its high antibiofilm activity, to observe the possible morphological changes on *E. coli* and MRSA. Bacteria were seeded on frosted glass coupons for 48 h. Micrographs obtained by SEM are shown in Figure 2 and Figure 3. Biofilm control of MRSA and *E. coli* O157:H7 in absence of EO are shown in micrographs A and B in each figure, while C and D correspond to the biofilm treated with the LOC EO (0.5 MIC_50_).

Figure 2A, shows an abundant formation of MRSA biofilm on the glass surface (3000×). In addition, Figure 2B shows a magnification (12,000×) of the previous image, where the spherical and smooth morphology of MRSA is clearly seen with an average bacterial size of 0.89 µm. Figure 3C, corresponds to the biofilm of MRSA treated with the EO and shows a significant decrease in the biofilm formation (3000×). By magnifying the previous image (12,000×, Figure 2D), the extracellular matrix of MRSA polysaccharide along with some bacteria can be observed, it has an irregular morphology and an average size of 0.68 µm, which is 23% smaller than that of the control group. 

As shown, in Figure 3A, a low biofilm formation of *E. coli* O157:H7 on the glass surface was evidenced (2000×). Figure 3B shows a magnification (8000×) of the previous image, the elongated and smooth morphology characteristic of *E. coli*, with an average bacterial size of 1.74 ± 0.30 µm can be observed. Figure 3C corresponds to *E. coli* treated with the LOC EO no biofilm formation was observed (200×). By magnifying the previous image (8000×, see Figure 3D), a large amount of extracellular matrix of polysaccharide, and what it appears to be the remains of lysed bacteria, are observed due to the action of the EO. 

## 3. Discussion

In this work it was found that Gram-positive bacteria were more susceptible to EOs than Gram-negative bacteria, this is a characteristic that has been documented in other studies [37], and this may be due to Gram-negative cell wall does not allow for the entrance of hydrophobic molecules as readily as Gram-positive bacteria. Nazzaro et al. described in their study the effect of EO on pathogenic bacteria, a phenomena that occur at cellular level; thus, EOs are less able to affect the cell growth of the Gram-negative bacteria. Due to the wide variety of molecules present in the natural extracts, the antimicrobial activity of the EOs cannot be attributed to a single mechanism. The effects of EOs usually lead to the destabilization of the phospholipid bilayer, the destruction of the plasma membrane function and composition, the loss of vital intracellular components and the inactivation of enzymatic mechanisms. In some cases, EOs also alter membrane permeability by destroying the electron transport system, and a number of components of the EOs, such as carvone, thymol, and carvacrol, lead to an increase in the intracellular concentration of ATP, an event that is linked to the destruction of the microbial membrane. When antimicrobial compounds are present in the environment surrounding microorganisms, the bacteria may react by altering the synthesis of fatty acids and membrane proteins to modify the fluidity of the membrane. The hydrophobicity of the EOs and their components allow them to diffuse through the double lipid layer of the membrane. The EOs can alter both the permeability and function of membrane proteins. The alteration of membrane permeability and the defects in the transport of molecules and ions result in a “disbalance” within the microbial cell. This subsequently leads to cytoplasm coagulation, the denaturation of several enzymes and of cellular proteins and the loss of metabolites and ions [21]. 

Only two EOs obtained from LOT and LOC had a high antibacterial activity against both bacteria (Table 2), one of the common characteristics of these EOs was their high content of thymol and carvacrol (Table 1), which have been studied extensively because of their antifungal and antibacterial properties [38]. It has been determined that the antibacterial activity of thymol is greater against *E. coli*, *S. aureus*, *Pseudomonas aeruginosa,* and *Salmonella enterica* in comparison to those of carvacrol, *trans*-cinnamic acid, and eugenol [39,40]. These results obtained in our studies are consistent with those published by other authors, because EOs with highest antibacterial activity (Table 2) are oils richer in thymol content (LOC: 32.7% and LOT: 22.1%). 

Nevertheless, it was also observed that EO from TV, despite having a high content of thymol (23%), did not have the same antibacterial activity against both bacteria, being ineffective against MRSA (MIC_50_ > 3). In this regard, other authors suggest that the effect of a single compound may not be as effective in inhibiting the growth and formation of biofilm, as may be two or more compounds, posing a potential synergistic effect between the components of the same oil [41], as it is the case of carvacrol and thymol present in LOC and LOT which can enhance its activity against MRSA [24]. 

EOs from *Cymbopogon* spp showed antimicrobial activity against *E. coli* (CM, MIC_50_ = 1.4 mg/mL) and MRSA (CF, MIC_50_ = 2.4 mg/mL), which may be related to the high content of geranial (33%) in CF and geraniol (38.7%) in CM; these compounds have been studied by other authors due to their many biological activities useful as antibacterial, antifungal, pesticides, insecticides and anticancer [42,43,44,45,46,47]. It has also been shown that geranial is the compound with the highest antimicrobial activity in this plant genus, while other components such as geraniol and geranyl acetate play a secondary role [48]. However, in this work we observed a low antibacterial activity of the CF (geranial, 33%) against *E. coli*, while the activity of CM (geraniol 38.7%) was higher, which suggests that these compounds may have different action mechanisms that may be related to the bacterial cell wall conformation. Finally, EO from RO presented antibacterial activity against MRSA (MIC_50_ = 2.5 mg/mL), which has been previously documented by other authors, and it has even been shown that EO from RO has a specific inhibitory activity against *S. aureus* [49]. This may be due to the presence of α-Pinene (12.7%) which has shown to present inhibitory activity against MRSA in previous experiments [50].

In this work it was also possible to determine the great antibiofilm potential activity of the EOs from LOT, LOC and TV, which can be attributed to high content of aromatic monoterpenes (thymol and carvacrol) present in these EOs. These oils showed a significant bactericidal activity when analyzed separately [40,51,52]. Due to the hydrophobic nature of carvacrol and thymol; these compounds can interact with the lipid bilayer of the cytoplasmic membranes, causing the loss of integrity and the leakage of cellular material such as ions, ATP and DNA [53,54].

Another attribute of carvacrol and thymol is their ability to diffuse through the polysaccharide matrix of the biofilm and destabilize it thanks to its strong intrinsic antimicrobial properties [55]. Knowles et al. (2005) suggests that the continuous exposure of *S. aureus* to non-biocidal concentrations of carvacrol interrupts the normal development of the biofilm, preventing the accumulation of protein mass and stopping micro-colony stage [56]. Alternatively, these compounds could interact with the protein surfaces, leading to an alteration of the bacterial cell surface and compromising the initial binding phase to the surfaces [57].

Other Eos, such as EO from CM, showed an interesting antibiofilm activity, attributable to the presence of eugenol, which can interfere with bacterial mobility, adhesion, and biofilm formation in *E. coli* [58,59]. Finally, the EO from CO showed a good antibiofilm activity, despite this EO did not show an apparent antibacterial activity (MIC_50_ > 3 mg/mL and MBC > 3 mg/mL). These results are similar to those obtained by Lee et al., which indicates that EO from CO negatively regulates the expression of the HLA gene of α-hemolysin in *S. aureus* [60], which is necessary for the formation of *S. aureus* biofilm [61].

Despite the high antimicrobial and antibiofilm activities, the EOs at high concentrations presented cytotoxicity. Cytotoxicity of EOs from LOT, LOC, and TV may be due to their high content of thymol. It is necessary to highlight that thymol has a limited use in drugs due to its moderate cytotoxicity, which it has been demonstrated both in vitro in human and animal cells, and in vivo in animal studies [38,62,63,64]. On the contrary, EO from CF presented a cytotoxic activity very similar to that other essential oils, which may be because of its high content on citral (neral and geranial) which has been demonstrated in in vitro cytotoxic tests with Vero and other cell lines, such as HeLa and MCF-7 [65,66]. Similar results have also been reported in other species of the *Cymbopogon* genus, such as *C. citratus* and *C. nardus*, which is attributed to the high content of geranial and neral [66].

Finally, the biofilm of SARM treated with the EO showed a significant decrease in the formation of biofilm, this effect may be due to the activity of thymol on the lipid bilayer of the bacterial cell membrane, which can cause disturbances and permeabilization of bacterial cell membrane, with the consequent loss of cellular content, irregular morphology and decrease in size of the bacteria [40,67,68,69]. On the other hand, it is also worth noting a low formation of biofilm in *E. coli* on the glass coupon, which is consistent with the results obtained by Adetunji et al. (2012), where it is concluded that *E. coli* O157:H7 is not a bacterium with great capacity to form biofilm in comparison with other bacteria, such as *Salmonella* sp. [70]. It should be mentioned that in biofilm formation, hydrophobicity of the bacteria and the surface are important aspects [71]. In our study, a significantly lower biofilm formation was observed in glass, which may be due to its high hydrophobicity, which makes difficult the adhesion and subsequent formation of the biofilm [70].

## 4. Materials and Methods 

### 4.1. Materials

Bacterial culture media: Brain Heart Infusion Agar (BHI), Tripticase Soy Broth (TSB) and D-glucose ≥ 99.5% were purchased from Sigma-Aldrich (Milwaukee, WI, USA). For the cell viability assay, 3-(4,5-dimethylthiazol-2-yl)-2,5-diphenyltetrazole (MTT) bromide was used in Hank’s Balanced Salt Solution (HBSS).

### 4.2. Plant Material and Extraction

Aromatic plants used in this work (Table 1) were obtained in different areas of the State of Santander (Colombia) and cultivated in experimental plots located in the Pilot Agroindustrial Complex (CENIVAM, N 07° 08,442, WO 73° 06,960 977 above mean sea level (amsl)) at the Universidad Industrial de Santander (UIS, Bucaramanga, Santander, Colombia). Taxonomic characterization of the plants was carried out in the Institute of Natural Sciences of the Universidad Nacional de Colombia (UNAL, Bogotá, Colombia).

Extraction of the essential oils was carried out by hydro-distillation in a Clevenger type equipment adapted to a microwave heating system (Samsung, MS-1242zk), with an output power of 1600 W and a radiation frequency of 2.4 GHz. The plant material (200 g of each plant) was suspended in water (300 mL) in a 2 L balloon, which was connected to a Clevenger-type glass device, with a Dean-Stark distillation reservoir. The plant sample was heated by microwave irradiation in three consecutive series of 15 min (45 min total). Extracted essential oil was dried with anhydrous sodium sulfate, weighed, and stored in an amber colored flask at 4 °C. All extractions were done by triplicate (*n* = 3) [28]. 

### 4.3. EOs Analysis by Gas Chromatography-Mass Spectrometry (GC/MS)

The EOs were analyzed by gas chromatography (Agilent Technologies 6890N Series Network System, Palo Alto, CA, USA), coupled to mass spectrometry (Agilent Technologies MSD 5975 Inert XL). A fused-silica capillary column DB-5MS (60 m × 0.25 mm id × 0.25 μm d_f_), with a stationary phase of 5% -phenyl-poly (dimethylsiloxane), and a polar capillary column DB-WAX (60 mm × 0.25 mm id × 0.25 μm d_f_), with stationary phase of polyethylene glycol were used. GC oven temperatures were programmed differently for each column as follows: 45 (5 min) to 150 °C (2 min) at 4 °C/min, then at 250 °C (5 min) at 51 °C/min, and finally, at 275 °C (15 min) at 10 °C/min, for the DB-5MS column and 45 (5 min) up to 150 °C (3 min) at 3 °C/min, then at 220 °C (5 min) at 4 °C/min for the DB-WAX column. The temperatures of the transfer line, the ionization chamber, and the injection port were set at 285 °C, 230 °C, and 250 °C, respectively. Helium was used as a carrier of gas (99.995% AP gas, Linde, Bucaramanga, Colombia), with a constant volumetric flow of 1 mL/min. 

Mass spectra were obtained by electron ionization (EI) with electron energy of 70 eV. Mass spectra, total ionic currents (TIC) and extracted ion (EIC) were obtained with a quadrupole analyzer, by means of automatic radiofrequency scanning (full scan) in the mass range of *m/z* 40–350 (5, 5 spectra/s). Components of the essential oils were identified by comparison of their mass spectra, obtained by GC/MS, and linear retention indexes (LRI) in the two columns; polar and apolar ones, calculated using the homologous series of *n*-alkanes C10-C25 (Sigma-Aldrich, Milwaukee, WI, USA) and compared to the mass spectra from the MS-ChemStation G1701-DA data system, which include the WILEY, NIST 2014, and QUADLIB 2007 spectral libraries. In order to assess identification of components in essential oils and, the following standard substances were used: geranyl acetate (98%), aromadendrene (97%), benzyl benzoate, β-caryophyllene (98.5%), 1,8-cineol (99%), *p*-cymene (99%), α-copaene (90%), *trans*-farnesol (96%), geraniol (98%), hexanal (98%), α-humulene (96%), linalool (97%), (R)-(+)-limonene (97%), menthol (99%), (+)-menthone (98.5%), *trans*-nerolidol (85%), caryophyllene oxide (95%), α-pinene (98%). All chemical standards were acquired from Sigma-Aldrich (Milwaukee, WI, USA) [28].

### 4.4. Bacterial Strains 

*E. coli* O157:H7 ATCC and MRSA strains were acquired from Strain Collection of Pontificia Universidad Javeriana, Colombia and School of Bacteriology and Clinical Laboratory of the Universidad Industrial de Santander (UIS), respectively. Cell culture maintenance of these strains was carried out in BHI medium at 37 °C.

### 4.5. Determination of the Minimum Inhibitory Concentration (MIC) and Minimum Bactericidal Concentration (MBC)

Evaluation of MIC and MBC of the pathogenic bacteria *E. coli* O157:H7 and MRSA, was carried out following the broth microdilution protocol standardized in the GIBIM [34], which is based on the protocol of the Clinical and Laboratory Standards Institute (CLSI) document M100 S20 [72]. 

Values of MIC_50_ and MBC were defined as the minimum concentration of EO being able to inhibit 50% and totally inhibits bacterial growth, respectively [73]. Additionally, it was determined if the activity of EOs was bacteriostatic; it was considered bacteriostatic if the value of the MBC was four times higher than the value of the MIC (MBC/MIC > 4) [74]. 

For MIC determinations, a pre-inoculum was prepared from fresh culture of tested strains using TSB and TSB supplemented with 0.25% (*w*/*v*) glucose for *E. coli* O157:H7 and MRSA, respectively. The pre-inoculums were incubated during 12 h at 37 °C and 200 rpm, until reaching a bacterial concentration equivalent to 4.4–5 × 10^9^ CFU/mL, using the Mcfarland scale as a reference [75]. Inoculum was prepared from the pre-inoculum, taking it to a final volume of 10 mL with sterile medium, until obtaining an absorbance between 0.07 and 0.1 (equivalent to ~5 × 10^5^ CFU) [35].

Subsequently, bacterial growth kinetics was monitored in 96-well ELISA microplates (Bio-Rad, Imarck), where 100 μL of the bacterial inoculum was plated along with 100 μL of each EO in serial concentrations from 0.18 to 3 mg/mL for both strains. These microplates were incubated at 37 °C with constant stirring (200 rpm), and microbial growth was evaluated measuring the absorbance every hour up to complete 24 h using an ELISA microplate reader at 595 nm. Wells containing bacterial cultures without EO were used as negative controls.

MBC was determined once cell growth kinetics of the pathogenic strains were evaluated. Aliquots of 100 μL from each well containing different EO concentrations were taken and plated in 2 mL Eppendorf tubes containing 900 μL of BHI; subsequently, these tubes were incubated at 37 °C for 24 h. To corroborate the bactericidal effect, an aliquot of 10 μL was taken from each tube and was transferred to BHI agar plates at 37 °C for 24 h.

### 4.6. Inhibition of Biofilm Formation

Potential biofilm inhibition of EOs was evaluated in a 96-well U-bottom microplate until reaching a final volume of 200 μL. This was performed by adding 100 μL of each EO at different concentrations and 100 μL of bacterial culture (~5 × 10^5^ CFU/mL) in every well. Wells containing bacterial cultures without EO were used as negative controls, and the microplates were incubated at 37 °C for 48 h without agitation, allowing the adherence of bacteria to the surface [57]. 

After incubation, liquid content from the wells was removed, and the microplate was rinsed three times with sterile saline solution 0.9% in order to eliminate planktonic bacteria; then, microplates were dried in an oven at 60 °C for 45 min. Subsequently, each well was stained with 200 μL of crystal violet 0.4% and incubated at a room temperature for 15 min. Thereafter, microplates were rinsed three times with sterile saline solution 0.9% to remove excess of crystal violet and 200 μL of acetic acid 30% in ultra-pure water [76]. The liquid content of each well was transferred to a new flat bottom microplate and the absorbance was measured at 595 nm using an ELISA microplate reader (Bio-Rad, lmarck version 1.02.01, Hercules, CA, USA). Each assay was performed by triplicate (*n* = 3). Minimum biofilm inhibition concentration (MBIC_50_) was defined as the minimum concentration of EO that can inhibit biofilm formation by 50%. Finally, inhibition percentages of each EO were calculated using the following formula [77,78]:

$$Inhibition\;Percentage=\frac{OD\;negative\;control-OD\;Experimental}{OD\;negative\;control}\times 100$$

### 4.7. EO Cytotoxicity Assay in VERO Cell Line

EOs with the high antibacterial and antibiofilm activities were tested to determine their cytotoxicity in non-tumor Vero cell line (African green monkey kidney, ATCC No. CCL-81) following the MTT methodology as was described by Mosman [79]. Vero cell line was maintained in Eagle’s Minimum Essential Medium (EMEM) supplemented with 10% fetal bovine serum at 37 °C in 5% CO_2_. Then, 1 × 10^4^ cells/mL were seeded in a polystyrene microplate 96-well flat bottom and incubated for 24 h. These cells were treated with serial dilutions of EOs. After 48 h of incubation, the supernatant was discarded, and 200 μL of MTT (500 μg/mL in HBSS) was added to each well and incubated for 3 h, and then, the supernatant was discarded and 200 μL of di-methyl-sulfoxide (DMSO) was added to solubilize the formazan crystals inside the cells. Absorbance at 550 nm of each well was measured in a microplate reader (Multiskan™ GO microplate spectrophotometer, Thermo Fisher Scientific, Waltham, MA, USA). The wells with DMSO without cells were used as blank. Cytotoxic Concentration 50% (CC_50_) was defined as the concentration of the compound that reduces the viability of the cell line by 50% [80,81]. Finally, the Selectivity Index (SI) was calculated as the division of IC_50_ over MIC_50_ [66]. All experiments were performed by triplicate (*n* = 3). The results are presented as the mean ± standard deviation. Cell viability of Vero cells cultured without EOs was considered as 100%.

### 4.8. Visualization of the Morphological Alterations by Scanning Electron Microscopy (SEM)

Observations of the possible morphological changes of both bacteria was done by SEM, following the protocol of Singh et al. with some modifications [82]. Frosted glass coupons (10 × 15 × 2 mm) were used in order to facilitate the biofilm formation, which were deposited in a flat-bottomed 12 well microplate. Subsequently, 1500 μL of the bacterial inoculum in 1/100 dilution was added and incubated at 37 °C for 48 h, in presence and absence of the EOs [83]. After 48 h, these glass coupons were rinsed three times with sterile saline solution 0.9% to eliminate the planktonic cell. Then, they were fixed with 2.5% glutaraldehyde for 60 min and dehydrated with an isopropanol gradient (10–95%) for 10 min [84]. Coupons were coated with gold and observed by SEM using a Quanta 650 FEG scanning electron microscope (SEM, Thermo Fisher Scientific, Waltham, MA, USA), which was equipped with an Everhart Thornley ETD detector. SEM images were taken under the following conditions: High vacuum, acceleration voltage 15 kV, and magnification between 600× and 25,000×.

### 4.9. Data Analysis

All the experiments were performed in triplicate and one-way analysis of variance (ANOVA) was used to analyze the differences among the treatments. In all cases, the level of significance was 0.05. Assumption of normality and equally of variances of data was previously tested using Shapiro–Wilk and Levene’s test, respectively.

## 5. Conclusions

In the present study, EOs from both LOT and LOC demonstrated the highest antibacterial and antibiofilm activity against *E. coli* O157:H7 and MRSA. These results were corroborated by SEM, where the effect of EO from LOC on the morphology of both bacteria was observed, which caused alterations in the cell surface, and bacterial size reduction and cell lysis. On the other hand, cytotoxicity tests on the Vero cell line indicated a high cytotoxic activity; possibly due to the high content of thymol and other compounds present in the EO. However, cytotoxicity problems observed for these EOs can be overcome by nanoencapsulation or microencapsulation methods, which can be able to preserve the active components as well as increase the solubility in aqueous medium and decrease their cytotoxicity.

Further studies are needed to elucidate the action mechanisms of the essential oils on the bacterial cells under study. In our research group, proteomic and metabolomic analyses are in progress to unraveling the possible therapeutic targets of the EO on the bacterial biofilm formation.

## Figures and Tables

**Table 1:** Chemical composition of the major components (%) of essential oils: *C. flexuosus* (CF), *C. martini* (CM), *C. nardus* (CN), *Cananga odorata* (CO), *L. alba,* carvona chemotype (LACA), *L. alba* citral, chemotype (LACI), *L. origanoides* carvacrol, chemotype (LOC), *L. origanoides* phellandrene, chemotype (LOF), *Lippia origanoides* thymol chemotype (LOT), *Rosmarinus officinalis* (RO), *Swinglea glutinosa* (SG), *Salvia officinalis* (SO), *Satureja viminea* (SV), *Tagetes lucida* (TL), *Thymus vulgaris* (TV).

Compound	Type	CF	CM	CN	CO	LACA	LACI	LOC	LOF	LOT	RO	SG	SO	SV	TL	TV
**1-Isopropenyl-4-methyl-1-cyclohexane**	**OC**	-	-	-	-	-	-	-	-	-	-	-	-	24.4	-	-
**2,6-dimethyl-2,6-octadiene**	**OC**	-	-	6.1	-	-	-	-	-	-	-	-	-	-	-	-
**Benzyl acetate**	**OC**	-	-	-	10.3	-	-	-	-	-	-	-	-	-	-	-
**Geranyl acetate**	**OC**	0.5	1.3	-	-	-	-	-	-	-	-	-	-	-	-	-
**Thymyl acetate**	**OC**	-	-	-	-	-	-	-	-	3.9	-	-	-	-	-	-
**Carvacrol**	**OC**	-	-	-	-	-	-	18.8	-	10.7	-	-	-	-	-	-
**Thymyl methyl eter**	**OC**	-	-	-	-	-	-	-	-	4.6	-	-	-	-	-	-
**Thymol**	**OC**	-	-	-	-	-	-	32.7	-	22.1	-	-	-	-	-	23.0
**Benzyl benzoate**	**OC**	-	-	-	20.8	-	-	-	-	-	-	-	-	-	-	-
**Methyl benzoate**	**OC**	-	-	-	3.7	-	-	-	-	-	-	-	-	-	-	-
***Cis* Cinnamile acetate**	**OC**	-	-	-	5.4	-	-	-	-	-	-	-	-	-	-	-
**Camphene**	**MH**	-	-	-	-	-	-	-	2.6	-	7.7	-	-	-	-	-
**Limonene**	**MH**	-	-	-	-	29.0	3.9	-	7.2	-	-	-	-	-	-	-
***p*-Cymene**	**MH**	-	-	-	-	-	-	1.1	11.2	3.7	-	-	-	-	-	20.0
***trans-β*-Ocimene**	**MH**	-	1.9	-	-	-	-	-	-	-	-	-	-	-	-	-
***α*-Phellandrene**	**MH**	-	-	-	-	-	-	-	9.9	-	-	-	-	-	-	-
***α*-Pinene**	**MH**	-	-	-	-	-	-	-	2.3	-	12.7	2.6	-	-	-	-
***β*-Myrcene**	**MH**	-	-	-	-	-	-	-	1.5	-	-	-	-	-	0.9	-
***γ*-Terpinene**	**MH**	-	-	-	-	-	-	5.2	-	-	-	-	-	-	-	9.5
***cis*-Pulegol**	**MH**	-	-	-	-	-	-	-	-	-	-	-	-	7.1	-	-
***1,8*-Cineol**	**OM**	-	-	-	-	-	-	-	11.6	-	17.5	36.6	5.3	-	-	-
**Camphor**	**OM**	-	-	-	-	-	-	-	-	-	14.8	-	8.5	-	-	-
**Carvone**	**OM**	-	-	-	-	31.3	-	-	-	-	-	-	-	-	-	-
**Citronellal**	**OM**	-	-	11.6	-	-	-	-	-	-	-	-	-	-	-	-
**Geranial**	**OM**	33.0	-	-	-	-	27.0	-	-	-	-	-	-	-	-	-
**Geraniol**	**OM**	7.9	38.7	17.8	-	-	6.1	-	-	-	-	-	-	-	-	-
**Linalool**	**OM**	-	3.2	-	11.7	-	-	-	-	-	-	-	-	-	-	4.7
**Neral**	**OM**	24.5	-	-	-	-	15.4	-	-	-	-	-	-	-	-	-
**Piperitone**	**OM**	-	-	-	-	1.5	-	-	-	-	-	-	-	-	-	-
***β*-Citronellol**	**OM**	-	-	16.9	-	-	-	-	-	-	-	-	-	-	-	-
***cis*-Thujone**	**OM**	-	-	-	-	-	-	-	-	-	-	-	5.8	-	-	-
**Estragole**	**OM**	-	-	-	-	-	-	-	-	-	-	-	-	-	79.9	-
**Pulegone**	**OM**	-	-	-	-	-	-	-	-	-	-	-	-	11.1	-	-
***trans*-Thujone**	**OM**	-	-	-	-	-	-	-	-	-	-	-	20.4	-	-	-
**Germacrene *B***	**SH**	-	-	-	-	-	-	-	-	-	-	10.8	-	-	-	-
**Germacrene *D***	**SH**	-	-	-	-	12.2	-	-	-	-	-	15.4	-	-	-	-
***trans-β*-Caryophyllene**	**SH**	-	-	-	-	-	11.8	6.4	11.3	7.9	7.8	-	-	11.8	-	9.5
***α*-Humulene**	**SH**	-	-	-	-	-	-	-	6.2	-	-	-	9.8	-	-	-
***β*-Bourbonene**	**SH**	-	-	-	-	2.4	-	-	-	-	-	-	-	-	-	-
***trans*-Nerolidol**	**OS**	-	-	-	-	-	-	-	-	-	-	24.0	-	-	-	-
**Monoterpenic hydrocarbons (MH)**		-	1.9	-	-	29.0	3.9	6.3	34.7	3.7	20.4	2.6	-	7.1	0.9	29.5
**Oxygenated monoterpenes (OM)**		65.4	41.9	46.3	11.7	32.8	48.5	-	11.6	-	32.3	36.6	40.0	11.1	79.9	4.7
**Oxygenated Compounds (OC)**		0.5	1.3	6.1	40.2	-	-	51.5	-	41.3	-	-	-	24.4	-	23.0
**Sesquiterpene hydrocarbons (SH)**		-	-	-	-	14.6	11.8	6.4	17.5	7.9	7.8	26.2	9.8	11.8	-	9.5
**Oxygenated sesquiterpenes (OS)**		-	-	-	-	-	-	-	-	-	-	24.0	-	-	-	-
**TOTAL MAJOR COMPONENTS IDENTIFIED (%)**		65.9	45.1	52.4	51.9	76.4	64.2	64.2	63.8	52.9	60.5	89.4	49.8	54.4	80.8	66.7

**Table 2:** MIC_50_ and MBC of 15 EOs against E. coli O157:H7 and MRSA. values are expressed in mg/mL, with experiment performed in triplicate (*n* = 3).

Abbrev.	Species	*E. coli O*157:H7		MRSA	
		MIC_50_	MBC	MIC_50_	MBC
**LACA**	*L. alba* (carvona)	>3	>3	>3	>3
**LACI**	*L. alba* (citral)	>3	>3	>3	>3
**CN**	*C. nardus*	>3	>3	>3	>3
**CM**	*C. martini*	1.4	>3	>3	>3
**CF**	*C. flexuosos*	>3	>3	2.4	3
**LOT**	*L. origanoides* (thymol)	0.9	1.5	1.6	3
**LOC**	*L. origanoides* (carvacrol)	0.4	0.7	0.6	1.5
**LOF**	*L. origanoides* (phellandrene)	>3	>3	>3	>3
**RO**	*Rosmarinus offiicinalis*	>3	>3	>3	>3
**SO**	*Salvia officinalis*	>3	>3	>3	>3
**SG**	*Swinglea glutinosa*	>3	>3	>3	>3
**TL**	*Tagetes lucida*	>3	>3	>3	>3
**TV**	*Thymus vulgaris*	0.9	1.5	>3	>3
**SV**	*Satureja viminea*	>3	>3	>3	>3
**CO**	*Cananga odorata*	>3	>3	>3	>3

**Table 3:** Effect of different EOs on the formation of biofilm in *E. coli* O157:H7 and MRSA. The inhibition percentage was calculated with respect to the control group and corresponds to the highest concentration of EO tested.

*Species*	*E. coli* O157:H7		MRSA	
	MIBC_50_ (mg/mL)	Inhibition (%)	MIBC_50_ (mg/mL)	Inhibition (%)
*L. alba* (carvone)	>3	16.9	>3	0
*L. alba* (citral)	>3	12.9	>3	36.9
*C. nardus*	>3	21.0	>3	4.1
*C. martini*	1.12	55.0	>3	25.8
*C. flexuosos*	>3	36.2	1.83	78.71
*L. origanoides* (thymol)	0.45	70.3	1.2	82.61
*L. origanoides* (carvacrol)	0.19	73.9	0.07	81.09
*L. origanoides* (phellandrene)	>3	32.3	>3	38.15
*Rosmarinus offiicinalis*	>3	34.7	>3	25.15
*Salvia officinalis*	>3	29.3	>3	35.9
*Swinglea glutinosa*	>3	21.0	>3	6
*Tagetes lucida*	>3	49.4	>3	49.2
*Thymus vulgaris*	0.45	70.9	>3	39.3
*Satureja viminea*	>3	23.5	>3	54.5
*Cananga odorata*	>3	16.9	0,57	83.0

**Table 4:** Selectivity index (SI) of the EOs in *E. coli* O157:H7 (EC), SARM (SA).

SPECIES	CC_50_	SI	
		EC	SA
***C. martini***	0.72	-	0.51
***L. origanoides* (thymol)**	0.46	0.53	0.37
***L. origanoides* (carvacrol)**	0.65	2.05	1.00
***Thymus vulgaris***	0.73	0.74	-

## References

[1] Wang Z., Zhang H., Han J., Xing H., Wu M., Yang T. (2017). Deadly Sins of Antibiotic Abuse in China. Infect. Control. Hosp. Epidemiol..

[2] Hoelzer K., Wong N., Thomas J., Talkington K., Jungman E., Coukell A. (2017). Antimicrobial drug use in food-producing animals and associated human health risks: What, and how strong, is the evidence?. BMC Vet. Res..

[3] Prestinaci F., Pezzotti P., Pantosti A. (2015). Antimicrobial resistance: A global multifaceted phenomenon. Pathog. Glob. Health.

[4] World Health Organization (2014). Antimicrobial Resistance: Global Report on Surveillance Tomado de. http://apps.who.int/iris/bitstream/10665/112642/1/9789241564748_eng.pdf.

[5] Tacconelli E., Carrara E., Savoldi A., Harbarth S., Mendelson M., Monnet D.L., Pulcini C., Kahlmeter G., Kluytmans J., Carmeli Y. (2018). Discovery, research, and development of new antibiotics: The WHO priority list of antibiotic-resistant bacteria and tuberculosis. Lancet Infect. Dis..

[6] Ma Z., Bumunang E.W., Stanford K., Bie X., Niu Y.D., McAllister T.A. (2019). Biofilm Formation by Shiga Toxin-Producing *Escherichia coli* on Stainless Steel Coupons as Affected by Temperature and Incubation Time. Microorganisms.

[7] Centers for Disease Control and Prevention Prevention, (CDC) (2013). Antibiotic Resistance Threats in the United States, Tomado de. http://www.cdc.gov/drugresistance/threat-report-2013/pdf/ar-threats-2013-508.pdf.

[8] Bauer P.R., Sampathkumar P. (2017). Methicillin-Resistant *Staphylococcus aureus* Infection in ICU. Crit. Care Med..

[9] Weiner L.M., Webb A.K., Limbago B., Dudeck M.A., Patel J., Kallen A.J., Edwards J.R., Sievert D.M. (2016). Antimicrobial-Resistant Pathogens Associated With Healthcare-Associated Infections: Summary of Data Reported to the National Healthcare Safety Network at the Centers for Disease Control and Prevention, 2011–2014. Infect. Control. Hosp. Epidemiol..

[10] Zoja C., Buelli S., Morigi M. (2010). Shiga toxin-associated hemolytic uremic syndrome: Pathophysiology of endothelial dysfunction. Pediatr. Nephrol..

[11] Caprioli A., Scavia G., Morabito S. (2014). Public Health Microbiology of Shiga Toxin-Producing *Escherichia coli*. Microbiol. Spectr..

[12] Kadariya J., Smith T.C., Thapaliya D. (2014). *Staphylococcus aureus* and Staphylococcal Food-Borne Disease: An Ongoing Challenge in Public Health. BioMed Res. Int..

[13] Jamal M., Ahmad W., Andleeb S., Jalil F., Imran M., Nawaz M.A., Hussain T., Ali M., Rafiq M., Kamil M.A. (2018). Bacterial biofilm and associated infections. J. Chinese Med. Assoc..

[14] Mayton H.M., Marcus I.M., Walker S.L. (2019). *Escherichia coli* O157:H7 and Salmonella Typhimurium adhesion to spinach leaf surfaces: Sensitivity to water chemistry and nutrient availability. Food Microbiol..

[15] Ueda Y., Mashima K., Miyazaki M., Hara S., Takata T., Kamimura H., Takagi S., Jimi S. (2019). Inhibitory effects of polysorbate 80 on MRSA biofilm formed on different substrates including dermal tissue. Sci. Rep..

[16] Stewart P.S. (2002). Mechanisms of antibiotic resistance in bacterial biofilms. Int. J. Med. Microbiol..

[17] Singh R., Ray P., Das A., Sharma M. (2009). Role of persisters and small-colony variants in antibiotic resistance of planktonic and biofilm-associated Staphylococcus aureus: An in vitro study. J. Med. Microbiol..

[18] Cepas V., López Y., Muñoz E., Rolo D., Ardanuy C., Martí S., Xercavins M., Horcajada J.P., Bosch J., Soto S.M. (2019). Relationship Between Biofilm Formation and Antimicrobial Resistance in Gram-Negative Bacteria. Microb. Drug Resist..

[19] Fastenberg J.H., Hsueh W.D., Mustafa A., Akbar N.A., Abuzeid W.M. (2016). Biofilms in chronic rhinosinusitis: Pathophysiology and therapeutic strategies. World J. Otorhinolaryngol. Head Neck Surg..

[20] Potera C. (1999). Forging a link between biofilms and disease. Science.

[21] Nazzaro F., Fratianni F., De Martino L., Coppola R., De Feo V. (2013). Effect of essential oils on pathogenic bacteria. Pharmaceuticals.

[22] Bassolé I.H.N., Juliani H.R. (2012). Essential Oils in Combination and Their Antimicrobial Properties. Molecules.

[23] Ambrosio C.M.S., de Alencar S.M., de Sousa R.L.M., Moreno A.M., Da Gloria E.M. (2017). Antimicrobial activity of several essential oils on pathogenic and beneficial bacteria. Ind. Crops Prod..

[24] Bazargani M.M., Rohloff J. (2016). Antibiofilm activity of essential oils and plant extracts against *Staphylococcus aureus* and *Escherichia coli* biofilms. Food Control..

[25] Bakkali F., Averbeck S., Averbeck D., Idaomar M. (2008). Biological effects of essential oils—A review. Food Chem. Toxicol..

[26] Swamy M.K., Akhtar M.S., Sinniah U.R. (2016). Antimicrobial Properties of Plant Essential Oils against Human Pathogens and Their Mode of Action: An Updated Review. Evid. Based Complement. Altern. Med..

[27] Marchese A., Arciola C.R., Coppo E., Barbieri R., Barreca D., Chebaibi S., Sobarzo-Sánchez E., Nabavi S.F., Nabavi S.M., Daglia M. (2018). The natural plant compound carvacrol as an antimicrobial and anti-biofilm agent: Mechanisms, synergies and bio-inspired anti-infective materials. Biofouling.

[28] Stashenko E.E., Martínez J.R., Ruíz C.A., Arias G., Durán C., Salgar W., Cala M. (2010). Lippia origanoides chemotype differentiation based on essential oil GC-MS and principal component analysis. J. Sep. Sci..

[29] Andrade E.H.A., Alves C.N., Guimarães E.F., Carreira L.M.M., Maia J.G.S. (2011). Variability in essential oil composition of Piper dilatatum L.C. Rich. Biochem. Syst. Ecol..

[30] Hussain A.I., Anwar F., Nigam P.S., Ashraf M., Gilani A.H. (2010). Seasonal variation in content, chemical composition and antimicrobial and cytotoxic activities of essential oils from four Mentha species. J. Sci. Food Agric..

[31] Rajendrudu G., Rama Das V.S. (1983). Interspecific differences in the constituents of essential oils of Cymbopogon. Chem. Lett..

[32] Kakaraparthi P.S., Srinivas K.V.N.S., Kumar J.K., Kumar A.N., Rajput D.K., Anubala S. (2015). Changes in the essential oil content and composition of palmarosa (*Cymbopogon martini*) harvested at different stages and short intervals in two different seasons. Ind. Crops Prod..

[33] Stashenko E.E., Martínez J.R., Durán D.C., Córdoba Y., Caballero D. (2015). Estudio comparativo de la composición química y la actividad antioxidante de los aceites esenciales de algunas plantas del género Lippia (Verbenaceae) cultivadas en Colombia. Rev. Acad. Colomb. Ciencias Exactas Físicas y Nat..

[34] Paredes D., Ortiz C., Torres R. (2014). Synthesis, characterization, and evaluation of antibacterial effect of Ag nanoparticles against Escherichia coli O157:H7 and methicillin-resistant Staphylococcus aureus (MRSA). Int. J. Nanomed..

[35] Espinel-Ingroff A. (2004). Métodos Estandarizados por el CLSI Para el Estudio de la Sensibilidad a Los Antifúngicos (Documentos M27-A3, M38-A y M44-A). Revista Iberoamericana de micología de España.

[36] Prayong P., Barusrux S., Weerapreeyakul N. (2008). Cytotoxic activity screening of some indigenous Thai plants. Fitoterapia.

[37] Man A., Santacroce L., Jacob R., Mare A., Man L. (2019). Antimicrobial activity of six essential oils against a group of human pathogens: A comparative study. Pathogens.

[38] Marchese A., Orhan I.E., Daglia M., Barbieri R., Di Lorenzo A., Nabavi S.F., Gortzi O., Izadi M., Nabavi S.M. (2016). Antibacterial and antifungal activities of thymol: A brief review of the literature. Food Chem..

[39] Olasupo N.A., Fitzgerald D.J., Gasson M.J., Narbad A. (2003). Activity of natural antimicrobial compounds against *Escherichia coli* and *Salmonella enterica* serovar Typhimurium. Lett. Appl. Microbiol..

[40] Lambert R.J.W., Skandamis P.N., Coote P.J., Nychas G.J.E. (2001). A study of the minimum inhibitory concentration and mode of action of oregano essential oil, thymol and carvacrol. J. Appl. Microbiol..

[41] Sandasi M., Leonard C.M., Viljoen A.M. (2008). The effect of five common essential oil components on *Listeria monocytogenes* biofilms. Food Control..

[42] Kim J.M., Marshall M.R., Cornell J.A., Preston J.F., Wei C.I. (1995). Antibacterial Activity of Carvacrol, Citral, and Geraniol against *Salmonella typhimurium* in Culture Medium and on Fish Cubes. J. Food Sci..

[43] Pattnaik S., Subramanyam V.R., Kole C. (1996). Antibacterial and antifungal activity of ten essential oils in vitro. Microbios.

[44] Pandey M.C., Sharma J.R., Dikshit A. (1996). Antifungal Evaluation of the Essential Oil of *Cymbopogon pendulus* (Nees ex Steud.) Wats. cv. Praman. Flavour Fragr. J..

[45] Delespaul Q., de Billerbeck V.G., Roques C.G., Michel G., Marquier-Viñuales C., Bessière J.-M. (2000). The Antifungal Activity of Essential Oils as Determined by Different Screening Methods. J. Essent. Oil Res..

[46] Pedroso R., Ueda-Nakamura T., Prado Dias Filho B., Aparicio Garcia Cortez D., Elaine Ranieri Cortez L., Andres Morgado-Diaz J., Nakamura C. (2006). Biological activities of essential oil obtained from *Cymbopogon citratus* on Crithidia deanei. Acta Protozool..

[47] Simic A., Rančic A., Sokovic M.D., Ristic M., Grujic-Jovanovic S., Vukojevic J., Marin P.D. (2008). Essential Oil Composition of *Cymbopogon winterianus* and *Carum carvi* and Their Antimicrobial Activities. Pharm. Biol..

[48] Kakarla S., Ganjewala D. (2009). Antimicrobial Activity of Essential Oils of Four Lemongrass (*Cymbopogon flexuosus* Steud) Varieties. Med. Aromat. Plant. Sci. Biotechnol..

[49] Gitaari N., Kareru P., Githua M. (2019). Antimicrobial Potential of Pelargonium citrosum and Rosmarinus officinalis Essential Oils. Int. Res. J. Pure Appl. Chem..

[50] Dhar P., Chan P., Cohen D.T., Khawam F., Gibbons S., Snyder-Leiby T., Dickstein E., Rai P.K., Watal G. (2014). Synthesis, Antimicrobial Evaluation, and Structure–Activity Relationship of α-Pinene Derivatives. J. Agric. Food Chem..

[51] Juven B.J., Kanner J., Schved F., Weisslowicz H. (1994). Factors that interact with the antibacterial action of thyme essential oil and its active constituents. J. Appl. Bacteriol..

[52] Ultee A., Gorris L.G., Smid E.J. (1998). Bactericidal activity of carvacrol towards the food-borne pathogen *Bacillus cereus*. J. Appl. Microbiol..

[53] Kumar A., Kamal A., Singh S., Padalia R.C., Tandon S., Chauhan A., Saikia D., Verma R.S. (2019). Chemical composition, antimicrobial activity, kinetics and mechanism of action of Himalayan-thyme (*Thymus linearis* Benth.). J. Essent. Oil Res..

[54] Martínez-Graciá C., González-Bermúdez C.A., Cabellero-Valcárcel A.M., Santaella-Pascual M., Frontela-Saseta C. (2015). Use of herbs and spices for food preservation: Advantages and limitations. Curr. Opin. Food Sci..

[55] Ouhayoun J.-P. (2003). Penetrating the plaque biofilm: Impact of essential oil mouthwash. J. Clin. Periodontol..

[56] Knowles J.R., Roller S., Murray D.B., Naidu A.S. (2005). Antimicrobial Action of Carvacrol at Different Stages of Dual-Species Biofilm Development by *Staphylococcus aureus* and *Salmonella enterica* Serovar Typhimurium. Appl. Environ. Microbiol..

[57] Nostro A., Roccaro A.S., Bisignano G., Marino A., Cannatelli M.A., Pizzimenti F.C., Cioni P.L., Procopio F., Blanco A.R. (2007). Effects of oregano, carvacrol and thymol on *Staphylococcus aureus* and *Staphylococcus epidermidis* biofilms. J. Med. Microbiol..

[58] Stratakos A.C., Linton M., Ward P., Campbell M., Kelly C., Pinkerton L., Stef L., Pet I., Stef D., Iancu T. (2019). The Antimicrobial Effect of a Commercial Mixture of Natural Antimicrobials Against *Escherichia coli* O157:H7. Foodborne Pathog. Dis..

[59] Pérez-Conesa D., McLandsborough L., Weiss J. (2006). Inhibition and inactivation of *Listeria monocytogenes* and *Escherichia coli* O157:H7 colony biofilms by micellar-encapsulated eugenol and carvacrol. J. Food Prot..

[60] Lee K., Lee J.H., Kim S.I., Cho M.H., Lee J. (2014). Anti-biofilm, anti-hemolysis, and anti-virulence activities of black pepper, cananga, myrrh oils, and nerolidol against *Staphylococcus aureus*. Appl. Microbiol. Biotechnol..

[61] Caiazza N.C., Toole G.A.O. (2003). Alpha-Toxin Is Required for Biofilm Formation by *Staphylococcus aureus*. J. Bacteriol..

[62] Manabe A., Nakayama S., Sakamoto K. (1987). Effects of essential oils on erythrocytes and hepatocytes from rats and dipalmitoyl phosphatidylcholine-liposomes. Jpn. J. Pharmacol..

[63] Suzuki Y., Furuta H. (1988). Stimulation of guinea pig neutrophil superoxide anion-producing system with thymol. Inflammation.

[64] Suzuki Y., Nakamura S., Sugiyama K., Furuta H. (1987). Differences of superoxide production in blood leukocytes stimulated with thymol between human and non-human primates. Life Sci..

[65] Mesa-Arango A.C., Montiel-Ramos J., Zapata B., Durán C., Betancur-Galvis L., Stashenko E. (2009). Citral and carvone chemotypes from the essential oils of Colombian *Lippia alba* (Mill.) N.E. Brown: Composition, cytotoxicity and antifungal activity. Mem. Inst. Oswaldo Cruz.

[66] Stone S., Vasconcellos F., Lenardão E., do Amaral R., Jacob R., Leivas Leite F. (2013). Evaluation of potential use of *Cymbopogon* sp. essential oils, (R)-citronellal and N-citronellylamine in cancer chemotherapy. Int. J. Appl. Res. Nat. Prod..

[67] Helander I.M., Alakomi H.-L., Latva-Kala K., Mattila-Sandholm T., Pol I., Smid E.J., Gorris L.G.M., von Wright A. (1998). Characterization of the Action of Selected Essential Oil Components on Gram-Negative Bacteria. J. Agric. Food Chem..

[68] Trombetta D., Castelli F., Sarpietro M.G., Venuti V., Cristani M., Daniele C., Saija A., Mazzanti G., Bisignano G. (2005). Mechanisms of Antibacterial Action of Three Monoterpenes. Antimicrob. Agents Chemother..

[69] Ultee A., Smid E.J. (2001). Influence of carvacrol on growth and toxin production by *Bacillus cereus*. Int. J. Food Microbiol..

[70] Adetunji V.O., Odetokun I.A. (2012). Assessment of Biofilm in *E. coli* O157:H7 and Salmonella Strains: Influence of Cultural Conditions. Am. J. Food Technol..

[71] Donlan R.M. (2002). Biofilms: Microbial Life on Surfaces. Emerg. Infect. Dis..

[72] CLSI (2017). Performance Standards for Antimicrobial Susceptibility Testing.

[73] Owuama C. (2018). Determination of minimum inhibitory concentration (MIC) and minimum bactericidal concentration (MBC) using a novel dilution tube method. Afr. J. Microbiol. Res..

[74] Pankey G.A., Sabath L.D. (2004). Clinical Relevance of Bacteriostatic versus Bactericidal Mechanisms of Action in the Treatment of Gram-Positive Bacterial Infections. Clin. Infect. Dis..

[75] García Rico R.O., Herrera Arias F.C. (2007). Evaluación de la inhibición del crecimiento de cinco cepas bacterianas patógenas por extractos acuosos de *Allium sativum*, *Allium fistulosum* y *Allium cepa*: Estudio preliminar in vitro. Bistua Rev. La Fac. Ciencias Básicas.

[76] Merritt J.H., Kadouri D.E., O’Toole G.A. (2005). Growing and Analyzing Static Biofilms. Current Protocols in Microbiology.

[77] Kifer D., Mu V., Maja Š. (2016). Antimicrobial potency of single and combined mupirocin and monoterpenes, thymol, menthol and 1, 8-cineole against *Staphylococcus aureus* planktonic and bio fi lm growth. J. Antibiot..

[78] Schillaci D., Arizza V., Dayton T., Camarda L., Di Stefano V. (2008). In vitro anti-biofilm activity of *Boswellia* spp. oleogum resin essential oils. Lett. Appl. Microbiol..

[79] Mosmann T. (1983). Rapid colorimetric assay for cellular growth and survival: Application to proliferation and cytotoxicity assays. J. Immunol. Methods.

[80] IUPAC (1997). Compendium of Chemical Terminology.

[81] Hennebelle T., Sahpaz S., Joseph H., Bailleul F. (2008). Ethnopharmacology of *Lippia alba*. J. Ethnopharmacol..

[82] Singh A., Gupta R., Tandon S., Pandey R. (2018). Anti-biofilm and anti-virulence potential of 3,7-dimethyloct-6-enal derived from *Citrus hystrix* against bacterial blight of rice caused by *Xanthomonas oryzae* pv. oryzae. Microb. Pathog..

[83] Atshan S.S., Shamsudin M.N., Than L., Lung T., Sekawi Z., Ghaznavi-rad E., Pei C.P. (2012). Comparative Characterisation of Genotypically Different Clones of MRSA in the Production of Biofilms. J. Biomed. Biotechnol..

[84] Singh V.K., Mishra A., Jha B. (2017). Anti-quorum Sensing and Anti-biofilm Activity of Delftia tsuruhatensis Extract by Attenuating the Quorum Sensing-Controlled Virulence Factor Production in *Pseudomonas aeruginosa*. Front. Cell. Infect. Microbiol..

